# Perioperative Challenges in a Patient With Moderate Congenital Factor VII Deficiency: A Case Report

**DOI:** 10.7759/cureus.96774

**Published:** 2025-11-13

**Authors:** Tahira El Ansari, Hassane Mamad, Souad Benkirane, Azlarab Masrar

**Affiliations:** 1 Hematology, Ibn Sina University Hospital, Mohammed V University of Rabat, Rabat, MAR

**Keywords:** bleeding risk, congenital coagulopathy, factor vii deficiency, perioperative management, rfviia

## Abstract

Congenital factor VII (FVII) deficiency is a rare autosomal recessive bleeding disorder characterized by marked clinical variability. The correlation between plasma FVII activity and bleeding severity is often poor, posing challenges in perioperative management. We report the case of a 25-year-old man with moderate congenital FVII deficiency (FVII:C 39%), diagnosed in infancy. He experienced severe bleeding after a dental extraction in 2008 but later underwent ileocecal resection in 2023 under recombinant activated factor VII (rFVIIa) coverage without complications. Preoperative laboratory investigations showed prolonged prothrombin time (PT 57% activity) with normal activated partial thromboplastin time (aPTT ratio 1.14), confirming isolated moderate FVII deficiency. A personalized perioperative protocol including rFVIIa (15-25 µg/kg IV), tranexamic acid, and local hemostatic measures was implemented. The patient tolerated the procedure well, with no postoperative bleeding or thrombosis. This case highlights that even moderate FVII deficiency can carry significant surgical bleeding risk, and emphasizes the importance of tailored perioperative management combining rFVIIa, antifibrinolytic therapy, and meticulous local hemostasis.

## Introduction

Congenital factor VII (FVII) deficiency is an inherited autosomal recessive disorder, first described by Alexander et al. in 1951 [1]. It is one of the rarest coagulation defects, with an estimated prevalence of approximately one in 500,000 individuals worldwide [2]. The deficiency results from quantitative or qualitative abnormalities of FVII, a vitamin K-dependent glycoprotein synthesized by the liver and essential for initiating the extrinsic coagulation pathway through its interaction with tissue factor (TF) [3,4].

The condition exhibits striking clinical heterogeneity, with a poor correlation between plasma FVII activity and bleeding severity. Some patients with activity levels below 5% may remain asymptomatic, whereas others with moderately reduced levels can experience significant hemorrhagic events, particularly during mucosal or visceral surgery where fibrinolytic activity is high [5-7]. This variability makes perioperative management particularly challenging and highlights the importance of individualized hemostatic assessment and tailored replacement therapy for safe surgical outcomes.

Standard perioperative management strategies for FVII deficiency include the use of recombinant activated factor VII (rFVIIa), antifibrinolytic agents such as tranexamic acid, and meticulous local hemostatic measures, with doses and intervals adjusted according to the patient’s bleeding history and laboratory response. However, data on optimal perioperative management in patients with moderate FVII deficiency remain limited, as most published cases focus on severe forms.

In this report, we describe the case of a 25-year-old Moroccan man with moderate congenital FVII deficiency. Despite relatively preserved FVII activity, he experienced severe postoperative bleeding following a minor dental extraction, underscoring the need for personalized perioperative planning, multidisciplinary coordination, and vigilant laboratory monitoring in such patients.

## Case presentation

A 25-year-old male, diagnosed at eight months of age with FVII deficiency, presented for preoperative assessment before dental extraction. There was no bleeding during circumcision. However, at the age of eight, he experienced severe bleeding after a dental extraction that required seven days of hospitalization and hemostatic therapy with topical fibrin sealant. The patient also reported a history of recurrent epistaxis and spontaneous bruising during adolescence, without major bleeding episodes in adulthood.

In April 2023, the patient underwent an ileocecal resection with perioperative rFVIIa (NovoSeven®, Novo Nordisk, Bagsværd, Denmark; 10 µg/kg every six hours for five days) and had an uneventful recovery. In 2024, he sought clearance for another dental procedure. Physical examination was normal with no active bleeding.

Preoperative laboratory investigations showed a prolonged prothrombin time (PT 57% activity) and a normal activated partial thromboplastin time (aPTT ratio 1.14), confirming isolated moderate FVII deficiency (see Table 1).

**Table 1:** Laboratory findings Laboratory test results showing an isolated moderate reduction in factor VII activity (39%) associated with prolonged PT (57% activity) and normal aPTT (ratio 1.14). Hemoglobin, platelet, and leukocyte counts were within normal limits, confirming the absence of anemia or other coagulation abnormalities. PT: prothrombin time; aPTT: activated partial thromboplastin time; FVII:C: factor VII coagulant activity; TCA: activated partial thromboplastin time

Test	Result	Reference Range
Hemoglobin	13.5 g/dL	13-17 g/dL
Platelets	299 × 10^9^/L	150-400 × 10^9^/L
Leukocytes	7.41 × 10^9^/L	4-10 × 10^9^/L
PT	57% activity	70-100%
aPTT (TCA, ratio)	1.14	<1.2
FVII:C	39%	50-145%

A personalized perioperative protocol including rFVIIa (15-25 µg/kg IV), tranexamic acid, and local hemostatic measures was implemented (see Table 2). Dose adjustments of rFVIIa were guided by the patient’s clinical response and laboratory parameters, particularly FVII activity, PT, and aPTT values. The patient tolerated the procedure well, with no postoperative bleeding or thrombotic complications.

**Table 2:** Perioperative management protocol Summary of the individualized perioperative management plan, including rFVIIa dosing schedule, antifibrinolytic therapy with tranexamic acid, use of local hemostatic agents, and postoperative monitoring strategy. rFVIIa: recombinant activated factor VII; PT: prothrombin time; aPTT: activated partial thromboplastin time; FVII:C: factor VII coagulant activity

Step/Measure	Description	Purpose/Outcome
Preoperative evaluation	Complete blood count, PT (% activity), aPTT (ratio), and FVII:C assay performed one week before surgery	Baseline assessment of coagulation status
Hemostatic replacement	Recombinant activated factor VII (rFVIIa, NovoSeven®) 10 µg/kg IV every six hours for five days, starting 30 minutes before incision	Maintain adequate FVII activity and ensure intra- and postoperative hemostasis
Antifibrinolytic therapy	Tranexamic acid 1 g IV every eight hours, continued for five days	Stabilize clot formation and prevent excessive fibrinolysis
Local hemostatic measures	Careful surgical technique, minimal tissue trauma, and use of topical fibrin sealant at the surgical site	Reinforce local hemostasis
Postoperative monitoring	Daily clinical examination and PT/aPTT measurement; assess for bleeding or thrombosis	Ensure hemostatic stability and detect complications early

## Discussion

FVII deficiency is a rare autosomal recessive bleeding disorder that presents with a highly variable clinical phenotype, ranging from asymptomatic laboratory abnormalities to life-threatening hemorrhages [4,5]. The clinical heterogeneity is largely explained by the diversity of mutations in the F7 gene and by the influence of additional genetic or environmental factors, such as TF expression, platelet function, or concomitant coagulation abnormalities [4,6].

**Figure 1:**
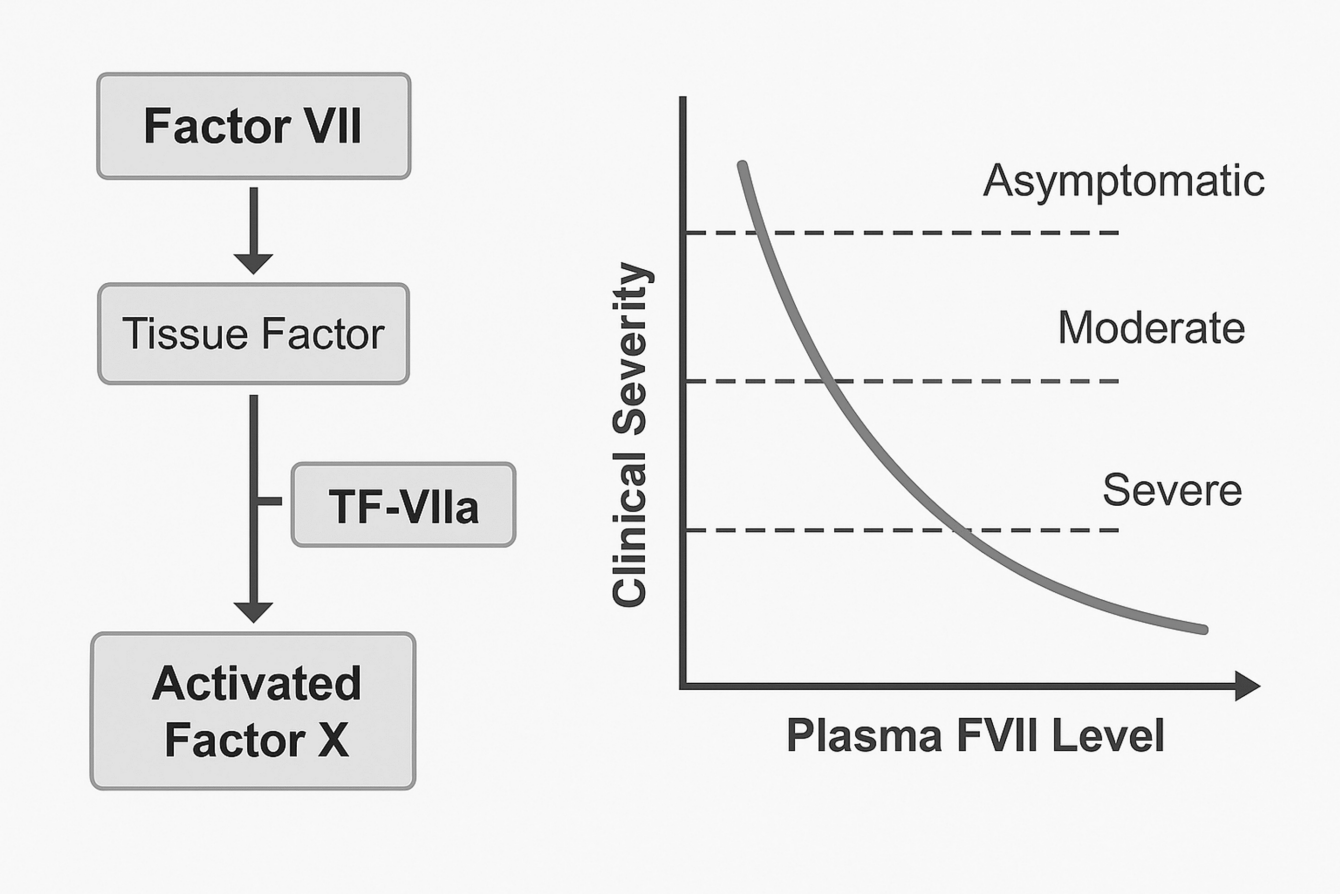
Role of factor VII (FVII) and tissue factor (TF) in the extrinsic coagulation pathway Diagram showing the position of factor VII (FVII) in the extrinsic coagulation pathway and the relationship between plasma activity levels and bleeding severity. Severe deficiency (<5%) results in spontaneous bleeding; moderate deficiency (30-50%) is associated with surgical or post-traumatic bleeding; mild deficiency (>50%) is often asymptomatic. Credit: Schematic created by the authors.

The extrinsic coagulation pathway, illustrated in Figure 1, highlights the crucial interaction between FVII and TF in the initiation of thrombin generation. This mechanism underscores why even moderate FVII deficiency can predispose patients to significant perioperative bleeding risk.

In clinical practice, there is often a poor correlation between plasma FVII activity and bleeding severity. Several studies have reported that patients with similar FVII levels may display markedly different bleeding tendencies, suggesting that FVII:C levels alone are not reliable predictors of clinical risk [6,7]. This variability complicates the perioperative management of affected individuals, particularly during major or mucosal surgeries where fibrinolytic activity is increased.

rFVIIa (NovoSeven®) remains the treatment of choice for both prophylactic and therapeutic management in congenital FVII deficiency. It acts by directly activating factor X in the presence of TF or on the platelet surface, bypassing the intrinsic pathway [8]. The optimal dosing regimen, however, varies among patients and procedures. Reported perioperative protocols recommend rFVIIa at doses of 15-30 µg/kg every four to six hours, with careful laboratory and clinical monitoring to balance efficacy and thrombotic safety.

These findings were compared with previous studies, which highlight the variability in clinical severity and emphasize the importance of individualized perioperative management. In our case, the patient tolerated surgery well under rFVIIa coverage without perioperative complications, confirming that short-term replacement therapy combined with antifibrinolytics and local hemostatic measures can provide adequate hemostasis even in moderate deficiency. These observations align with previous reports indicating that individualized therapy, based on prior bleeding history and procedural risk, remains the cornerstone of management [7,9].

Despite the rarity of congenital FVII deficiency, awareness of its variable presentation is essential for clinicians involved in perioperative care. A multidisciplinary approach involving hematologists, anesthesiologists, and surgeons ensures optimal outcomes by integrating laboratory data, clinical assessment, and appropriate use of replacement therapy.

## Conclusions

This case highlights that even moderate FVII deficiency can pose significant perioperative bleeding risks. Individualized hemostatic planning, including rFVIIa replacement, antifibrinolytic therapy, and close clinical monitoring, is essential for ensuring safe surgical outcomes. Awareness and multidisciplinary coordination remain key to optimizing management in patients with this rare disorder. However, as this report describes a single case, further multicenter or registry-based studies are needed to validate and generalize these findings.
